# The Role of Body Image Factors in Swedish Adolescents’ Sexting Behaviors

**DOI:** 10.1007/s10508-025-03115-4

**Published:** 2025-03-19

**Authors:** Jonas Burén, Kristina Holmqvist Gattario, Carolina Lunde

**Affiliations:** Department of Psychology, University of Gothenburg, Box 100, 405 30 Gothenburg, Sweden

**Keywords:** Sexting, Adolescents, Body image, Pubertal timing, Gender, Body surveillance

## Abstract

The current study tested a model in which three body image factors were assumed to be associated with adolescents’ sexting (sending nude or semi-nude pictures and/or video clips) to either romantic partners or strangers. We hypothesized that lower appearance esteem, more body surveillance, and more dysfunctional appearance beliefs would be associated with a higher likelihood of sexting. We also examined the effects of pubertal timing in the model, along with assessing the moderating effect of gender on the pathways to sexting. A questionnaire study was conducted with 1543 adolescents (girls = 791, 50.7%; boys = 772, 49.3%), ages between 13 and 16 years. The results from structural equation modeling indicated that girls’ and boys’ dysfunctional appearance beliefs were most consistently associated with sexting with romantic partners and strangers. Body surveillance also predicted sexting with strangers, but only for boys. Contrary to our hypotheses, appearance esteem was not significantly associated with sexting. Early-maturing girls and boys were more likely to sext with either partner. Girls reporting earlier pubertal timing were more likely to sext with romantic partners via the mediation of appearance beliefs. The findings from this study provide novel evidence of the complex interplay between body image and adolescents’ sexting, emphasizing that, in general, dysfunctional appearance beliefs seem to be more important for adolescents’ sexting compared to body surveillance and appearance esteem.

## Introduction

In recent years, adolescents’ social landscape has been fundamentally transformed by rapid digitalization. Adolescents are using instant messaging, social networking sites, and online games to interact with others. The digital sphere has also become an arena where adolescents engage in different sexual activities by searching for information about sex, chatting about sex, watching porn, or by sexting. Although the original definition of sexting used to emphasize text-based sexual interactions, the increased reliance on visual content for digital interaction has meant that sexting is increasingly considered a visual-based practice that involves the sexual portrayal of one's body to others (Mori et al., 2022). This shift is also reflected in the current article’s definition of the phenomena, as we solely focus on sexting as the sending of self-produced sexually explicit nude or semi-nude images/videos (Barrense-Dias et al., 2017, 2019; Lenhart, 2009; Ringrose et al., 2012). Contemporary research stresses sexting as a normative part of adolescents’ healthy sexual development, providing positive opportunities for adolescents’ sexual discovery, sexual self-expression, and intimacy enhancement (Steinberg et al., 2019). Yet, it should be recognized that sexting comes with risks too, such as non-consensual or pressured sexting.

Although it has been suggested that adolescents’ body image, referring to one's thoughts and feelings about one's body and appearance (Grogan, 2016), is an important driver of sexting behavior (e.g., Bianchi et al., 2017; Howard et al., 2021; Liong & Cheng, 2019; Speno & Aubrey, 2019), it remains poorly understood how different aspects of body image is related to adolescent sexting. This lack of knowledge is unfortunate since body image becomes an increasingly important aspect of adolescents’ views of themselves as they begin to navigate their emerging sexual self. Although most adolescents report feeling comfortable with their body and appearance (Public Health Agency of Sweden, 2023), numerous studies show that many adolescents struggle with body image concerns (Lacroix et al., 2023). For these adolescents, it has been suggested that sexting may be used as a tool to validate one’s body adequacy and to feel more comfortable in one’s body (Bianchi et al., 2016; Howard et al., 2021). Despite this, Howard et al. (2021) recently argued that "body image as a correlate of sexting patterns has received minimal interest in the literature” (p. 2). Very few studies have examined the links between body image and sexting in adolescent populations, despite the accentuated developmental importance of body image during this time. The current study aims to investigate the links between different body image factors and adolescents’ sexting behaviors. To this end, we analyzed data collected from a sample of 1543 mid-adolescents (age 13–16), who were queried about their body image and sexting behaviors. In the next section, we will provide the rationale for the current study. It has been based upon considerations of the developmental transitions that occur in adolescence, which fuel sexual development and lead to body image change. We will then outline the theoretical (the integrative cognitive-behavioral perspective; Cash, 2012) and empirical evidence that informed the propositions of this study.

### Understanding Sexting in the Context of Adolescent Developmental Change

The role of body image in adolescent sexting should be situated within the context of the dramatic biological, cognitive, and psychosexual changes occurring in early- and mid-adolescence (Steinberg, 2011). These changes are driven by pubertal development, which makes adolescents increasingly motivated to explore their sexuality and engage in sexual activities (Diamond & Savin-Williams, 2009). Questions of sexuality also become more salient in the adolescents’ immediate social context: among peers, in school, and through media and the internet (Temple-Smith et al., 2016). Adolescents need to figure out their own sexual self while navigating a social landscape immersed in normative notions and messages about sexuality (e.g., gendered sexual double standards; Crawford & Popp, 2003). Furthermore, the physical transformations associated with puberty draw attention to the body, appearance, and issues of attractiveness (Burgess et al., 2006; Lindberg et al., 2007). The physical and sexual changes associated with puberty drive body image change (McCabe & Ricciardelli, 2004). Studies further show that pubertal timing (i.e., developing early, on time, or late) is associated with both adolescents’ body image (Hamlat et al., 2015; Stice, 2003; Stojković, 2013; Williams & Currie, 2000) and sexual behaviors (see e.g., Skoog et al., 2009). For example, off-time pubertal maturation, particularly early maturation, has been associated with greater body image concerns in girls (Williams & Currie, 2000) but more body satisfaction in boys (Frisén & Holmqvist, 2010). One study using retrospective reports showed that girls’ early development was associated with greater sex-appeal self-worth, but also body surveillance and less body appreciation (Grower et al., 2019). Using a sample of 14-year-old boys only, Skoog et al. (2009) showed that early developers engaged in more online sexual activities. However, and despite the evidence suggesting that pubertal development is intertwined with body image and sexual behaviors, the joint associations between pubertal timing, body image and adolescents’ sexting behaviors remain unexplored.

### Theoretical Underpinnings and State of the Art

To understand the role of body image for adolescent sexual behaviors, including sexting, we rely on the well-established integrative cognitive-behavioral perspective (Cash, 2012). Within the cognitive-behavioral tradition, scholars usually distinguish between body image evaluation and body image investment. Whereas body image evaluation refers to satisfaction (or dissatisfaction) with one’s body, body image investment refers to the cognitive and behavioral importance of the body for self-evaluation (Cash, 2012). The closely related construct appearance-schema (see e.g., Cash, 2012; Markus et al., 1987) was introduced to describe how individuals who are “appearance schematic” (as opposed to appearance-aschematic) place great importance on appearance which is reflected in their implicit attitudes and beliefs about the role of appearance in their lives. For example, an individual who is highly appearance schematic may hold beliefs that appearance is an important part of who they are, that appearance is responsible for what happens to them, and that they need to always look their best. Although much of the research originating from this perspective has had a focus on body image pathology, Cash (2012) stressed that it also allows for an understanding of adaptive and positive aspects of individual’s relationship with their bodies, and the role that the body-self relationship plays in different behaviors.

In this study, we explore three body image related factors and how they are associated with adolescents’ sexting behaviors. In line with the cognitive-behavioral perspective, the explored body image factors encompass both evaluative (appearance esteem) and investment (body surveillance and dysfunctional appearance beliefs) aspects of body image. First, we argue that appearance esteem, which refers to evaluations of one’s physical appearance, may be associated with adolescents’ engagement in sexting. Research investigating adolescents’ motives for sexting suggests that one of most common motives (apart from sexual ones) is that sexting is used to gain body image reinforcement by attracting positive feedback about the body (Bianchi et al., 2017, 2021). These findings suggest that adolescents who have low appearance esteem may engage in sexting as a means of seeking appearance validation. Although there are no studies investigating the links between appearance esteem and sexting in adolescent samples, there has been a few studies in support of the above assumption. For example, Bianchi et al. (2017) showed that body shame (a negative evaluation of the body) is associated with sexting (Bianchi et al., 2017). Another study, which focused on sexting behaviors over one week in a sample of adult women (Howard et al., 2019), identified a link between higher levels of state body dissatisfaction and feeling pressured to sext. Women who were dissatisfied with their appearance were also more likely to sext for body image reinforcement during the study. In yet another study, body esteem explained a significant proportion of the variance in emerging adult women’s (23%) and men’s (35%) sexting behaviors (Howard et al., 2021). Although these studies seem to suggest that low appearance esteem may be a driver for engaging in sexting, it should also be recognized that an opposite direction of effect is also possible: It may also be that adolescents who have high appearance esteem may be the ones who are more likely to engage in sexting given that they may feel more comfortable displaying their bodies to others. Nevertheless, based on the results from previous studies, we propose that lower levels of appearance esteem are associated with an increased likelihood of engaging in sexting (H1).

Second, and in line with the theoretical framework (Cash, 2012), we propose two additional body image constructs that could be linked to adolescents’ sexting behaviors: body surveillance and dysfunctional appearance beliefs. These both mirror the investment aspects of body image. Body surveillance refers to the continual monitoring of one’s own body and is a key concept originating from the objectified body consciousness theoretical framework (see McKinley & Hyde, 1996). This perspective emphasizes that strict societal appearance norms together with sexual objectification of the (female) body cause individuals to feel as if their bodies are flawed and in need of constant monitoring (body surveillance). Relying on this theoretical perspective, it has been suggested that sexting can be viewed as a self-objectifying practice, as adolescents who engage in sexting have internalized a view of the body as a sexual object. The second investment construct in this study, dysfunctional appearance beliefs, refers to the cognitive belief that physical appearance has significant implications in everyday domains such as relationships, self-views, and emotions (Spangler & Stice, 2001). When individuals report high levels of dysfunctional appearance beliefs, they have an exaggerated perception that their appearance has significant importance in social situations. Higher dysfunctional appearance beliefs also mean equating one’s personal worth to physical appearance (Spangler & Stice, 2001). In line with the perspective of appearance-schemas (Cash, 2012), a high degree of body surveillance and dysfunctional appearance beliefs can be equaled to being appearance schematic. To date, a few studies have explored the associations between investment aspects of body image (such as body surveillance or dysfunctional appearance beliefs) and sexting. Speno and Aubrey (2019) showed that among 200 American adolescents, greater importance of physical appearance for self-concept was related to more favorable sexting attitudes in girls, whereas internalization of body ideals was related to more favorable sexting attitudes among boys. Bianchi et al. (2017) found in their study of older adolescents’ motives for engaging in sexting that objectified body consciousness (including body surveillance) was associated with sexting for sexual purposes. Similar results were found in Liong and Cheng’s (2019) study of college students. To our knowledge, no studies have examined the association between dysfunctional appearance beliefs and sexting, but it can be assumed that the belief that physical appearance is a central part of one’s relationships may be a predictor of adolescents’ sexting behavior. Based on these theoretical assumptions and empirical findings, we propose that body surveillance (H2) and dysfunctional appearance beliefs (H3) will be associated with a greater likelihood of engaging in sexting.

### The Role of Pubertal Timing for the Association Between Body Image and Sexting

As mentioned previously, evidence shows that pubertal timing is related both to adolescents' body image and their sexual behaviors. According to the maturational deviance hypothesis (Alsaker, 1995), adolescents who perceive themselves as being “off” relative to their peers (i.e., being early or late developers) often report more body image concerns. Although the literature testing the maturational deviance hypothesis has yielded inconclusive findings, the most consistent one is that early-maturing girls are more likely to have negative evaluations of their bodies (Hamlat et al., 2015; Williams & Currie, 2000). Early-maturing boys have been described as having both higher body esteem (McCabe & Ricciardelli, 2004) and lower body esteem (Rauste-von Wright, 1989). Moreover, studies have shown that both early-maturing girls and boys are more likely to engage in sexual activity and participate in risky sexual behaviors (Baams et al., 2015; Negriff et al., 2011; Skoog et al., 2013), as well as sexting (Burén & Lunde, 2018). With these findings in mind, earlier pubertal timing should be associated with a higher likelihood of engaging in sexting. Given the effect of pubertal timing on adolescents' body image, it is also possible that the association between pubertal timing and sexting is mediated by body image factors. That is, earlier pubertal timing will lead to more negative aspects of body image, which in turn might be associated with a higher likelihood of sexting (e.g., for appearance validation). Therefore, we propose that early pubertal timing will be associated with sexting both directly (H4) and via the mediation of body image (appearance esteem [H5a], body surveillance [H5b], and dysfunctional appearance beliefs [H5c]).

### Exploring the Role of Body Image for Sexting to Different Recipients

To further extend the literature on adolescents’ body image and their sexting behaviors, we argue that it might be important to distinguish between different kinds of recipients that adolescents may sext with. Sending sexually suggestive or nude images to a romantic partner is more common (Burén & Lunde, 2018), and it may be qualitatively different from sending those images to someone unknown. Indeed, “stranger danger” (the fear of young people being exposed to sexual predators) is a major concern among adults that has moved onto the digital landscape (Dedkova, 2015; Stokes, 2009). This fear has also permeated the public debate (and research for that matter), which tends to adopt a “risk frame” (Lippman & Campbell, 2014) of adolescent sexting. A recent study indicated that adolescents themselves generally accept the idea of sexting with a romantic partner, while they are more wary of the potential “stranger danger” online (Burén et al., 2022). Consequently, the threshold for adolescents to sext with someone unknown may be higher due to the perceived risk, and one may speculate that adolescents who sext with strangers could be more vulnerable in terms of issues such as body image. For instance, adolescents with body image concerns may use sexting to seek validation or appearance validation, where validation from unknown others might be perceived as more “genuine” because it comes from individuals who are not influenced by pre-existing relationships and biases. In their drive for appearance validation, adolescents might also be more inclined to extend their social networks to gain “fresh” feedback, which in turn makes them less perceptive of the risks associated with interacting with strangers online (Kim & Chock, 2015). A recent study showed that body image concerns among adolescent girls were positively related to intimate relationships with strangers they met online, which could also predict online sexual victimization (Longobardi et al., 2021). On the other hand, when sexting with a romantic partner, body image concerns might be less salient, and the decision to sext may be more driven by the desire to strengthen the bond or for sexual intimacy. Although it should be emphasized that it is not without risk to sext with a romantic partner, studies have also indicated that sexting with someone unknown may carry more risk for adolescents (e.g., Dowdell, 2011; Fleming & Rickwood, 2004; Livingstone et al., 2011; Rice et al., 2014; Williams et al., 2013). To date, no studies have explored the potential relationships between body image and sexting while also distinguishing between different types of sexting partners. However, due to the limited number of studies that make this distinction, we were unable to formulate recipient-specific hypotheses. Instead, the following explorative research question (RQ1) was used: Will the strength of the relationship between the body image factors (appearance esteem, body surveillance, and dysfunctional appearance beliefs) and sexting be different depending on whether the recipient is a romantic partner or stranger?

### Exploring the Role of Gender for the Association Between Body Image and Sexting

Finally, understanding the link between sexting and body image requires careful consideration of gender differences, as research suggests that gender plays a significant role in shaping individuals' experiences and perceptions in both domains. For example, girls experience more body image issues than boys, given that girls’ appearances are more critically surveilled by society, often leading to greater pressure on girls to conform to idealized standards of beauty (Fredrickson & Roberts, 1997; Wertheim et al., 2009). For sexting, research has shown gender differences in the prevalence, motivations, and consequences of sexting, such that girls are more likely to receive sexts, feel pressured to sext, and are more likely to be ashamed of it (e.g., Burén & Lunde, 2018; Burén et al., 2022; Mori et al., 2022; Van Ouytsel et al., 2017). Previous research on the relationship between body image factors and adolescents' sexting has shown different results concerning gender differences. Both Bianchi et al. (2017) and Liong and Cheng (2019) found no differences, while Speno and Aubrey (2019) found that more favorable sexting attitudes was associated with trait self-objectification among girls, and internalization of media ideals among boys. For these reasons, we will test the moderation of gender with the following research question (RQ2): Will gender moderate the association between pubertal timing, body image factors, and sexting between girls and boys?

### The Current Study

**Fig. 1:**
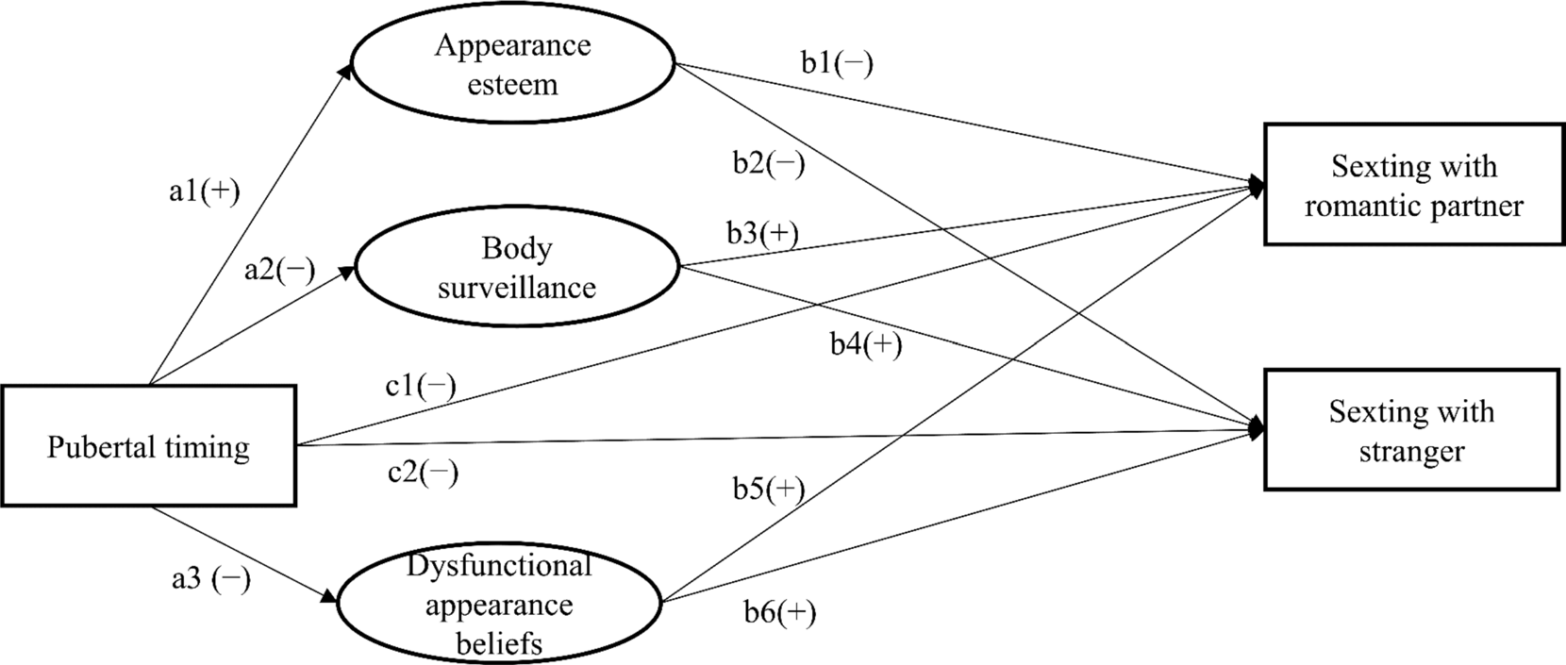
The hypothesized model. *Note.* H1, Appearance esteem (b1, b2) → Sexting; H2, Body surveillance (b3, b4) → Sexting; H3, Dysfunctional appearance beliefs (b5, b6) → Sexting; H4, Pubertal timing (c1, c2) → Sexting; H5a, Pubertal timing (a1) → Appearance esteem (b1, b2) → Sexting; H5b, Pubertal timing (a2) → Body surveillance (b3, b4) → Sexting; H5c, Pubertal timing (a3) → Dysfunctional appearance esteem (b5, b6) → Sexting. The plus or minus signs indicate expected positive or negative paths. The differences in the strength of the association between body image factors and the two sexting outcomes are explored for all paths (RQ1). The moderating effect of gender is explored for all paths to the sexting outcomes (RQ2)

Although body image related factors are potentially key variables in understanding adolescents’ sexting behaviors, to date, no studies have examined the association between body image and sexting while also distinguishing whom adolescents sext with (romantic partners and strangers). Previous studies have also had relatively small samples with most participants being late adolescents or young adults, implying that even less is known about the role of body image factors among younger adolescents who are in the midst of pubertal development. The present study was designed to examine the links between body image factors, pubertal timing and adolescents’ sexting behavior with a proposed model that evaluates the aforementioned hypotheses: H1, lower levels of appearance esteem are associated with an increased likelihood of engaging in sexting; H2, higher levels of dysfunctional appearance beliefs are associated with an increased likelihood of engaging in sexting; H3, higher levels of body surveillance are associated with an increased likelihood of engaging in sexting; H4, earlier pubertal timing is associated with an increased likelihood of engaging in sexting; H5a-c, this association will be mediated by the body image factors, with earlier pubertal timing being associated with higher levels of body image concerns (i.e., lower appearance esteem, more body surveillance, and more dysfunctional appearance beliefs) which is then associated with increased likelihood of engaging in sexting. In addition, we examined differences in the strength of associations between the body image factors and sexting with a romantic partner versus stranger (RQ1), as well as exploring the moderating role of gender (RQ2). A visualization of the hypothesized model and the expected paths can be found in Fig. 1.

## Method

### Participants and Procedure

The participants were recruited from 10 primary schools in Sweden. In compliance with Swedish laws, parental consent was obtained for presumed participants under the age of 15 before soliciting their participation in the study. Initially, we reached out to the legal guardians of these participants via regular mail, providing them with detailed information about the study along with a parental consent form. If we did not receive the completed consent form within a specified timeframe, we followed up by telephone to verbally inform the legal guardians about the study and obtain verbal consent. At least one legal guardian's consent was required before approaching the presumed participant to invite their participation. After obtaining parental consent, prior to each data collection session, we directly communicated with the participants. During this communication, we explained the purpose of the study, emphasized the voluntary nature of their participation, and assured them of their right to withdraw at any point. Additionally, we explicitly stated that they could decline to participate regardless of parental consent. For participants over the age of 15, we directly communicated with the participants and informed them of the purpose of the study, emphasized that their participation was voluntary, and assured them of their right to withdraw at any point during the study.

For each data collecting session, the participants filled in a questionnaire in a classroom setting during school time using a computer, tablet, or mobile phone. The data collection sessions were conducted one class at a time, with no more than 30 participants present for each session. Participants were seated separately in the classroom to prevent them from viewing each other's screens. Furthermore, each data collection session was gender-separated to reduce participant discomfort when responding to sensitive questions. The participants chose the gender group with which they identified. To ensure the integrity of the data collection process, at least one researcher and one teacher were present during each session.

Of the total enrolled students, 1673 (73.1%) adolescents, attending Swedish grades 7–9 (age range 12–16 years), completed the questionnaire. Of these, 110 participants did not respond to the key variables (sexting questions, gender, or pubertal timing) and were omitted from subsequent analyses. In addition, 20 participants did not indicate their gender or chose the third alternative (“other”). Due to the limited number of participants in this group, which would have hindered the formation of a group suitable for meaningful testing in subsequent statistical analyses, we chose to exclude these participants. This left a total of 1563 participants (50.7% girls and 49.3% boys) that were included in the analyses, *M* age = 14.19 (*SD* = 0.90).

### Measures

#### Demographic Questions

Gender was measured by asking the participants if they identified with being a girl or a boy, or other.

#### Appearance Esteem

The Appearance subscale of the Body Esteem Scale for Adolescents and Adults (BESAA; Mendelson et al., 2001) translated into Swedish (Erling & Hwang, 2004), was used to measure appearance esteem. This subscale has 10 items such as: “I’m pretty happy about the way I look”. The items are answered on a 5-point Likert scale ranging from 1 = *never* to 5 = *always*. Higher scores indicate higher appearance esteem. In the present sample, the subscale demonstrated good internal consistency (*α* = 0.89). In order to establish that the 10 items represent a stable latent variable, we conducted a one-factor CFA. Although previous evaluations of the Swedish BESAA show support for the appearance esteem items to reflect one factor (Kling et al., 2019), the present analysis indicated a poor fit (root mean square of approximation [RMSEA] = 0.193; comparative fit index [CFI] = 0.769; non-normed fit index [NNFI] = 0.704; standardized root mean square residual [SRMR] = 0.099). We reasoned this could be because four items in the subscale are reversed from the other six items (Brown, 2003). To correct for this potential issue, these items were allowed to correlate freely in our model, which still yielded a poor fitting model (RMSEA = 0.115, CFI = 0.933; NNFI = 0.896; SRMR = 0.037). We reasoned another potential issue could be that item 3 and 4 are too similar in meaning since both items ask about the wish to change one’s appearance: “There are a lot of things I’d change about my looks if I could” vs. “I wish I looked better”. Modification indices confirmed that these two items were problematic, and they were allowed to correlate freely. A final CFA indicated that with these corrections, the one-factor model had a more acceptable fit (RMSEA = 0.091, CFI = 0.959; NNFI = 0.934; SRMR = 0.035).

#### Body Surveillance

The body surveillance subscale from the Objectified Body Consciousness Youth Scale (Lindberg et al., 2006), previously translated into Swedish (Lunde & Frisén, 2011), was used in this study. The body surveillance subscale has four items, for example: “I often compare how I look with how other people look,” and is answered on a 7-point Likert scale ranging from 1 = *strongly disagree* to 7 = *strongly agree.* Higher scores indicate higher levels of body surveillance. In the present study, the subscale demonstrated high internal consistency (*α* = 0.91). For this subscale, we conducted a one-factor confirmatory factor analysis (CFA) to assess the appropriateness of using the four items to create a latent variable (“Body surveillance”). The CFA indicated an adequate fit (RMSEA = 0.103; CFI = 0.986; NNFI = 0.957; SRMR = 0.016).

#### Dysfunctional Appearance Beliefs

Nine items from the 20-item Dysfunctional Appearance Beliefs Scale (BAAS; Spangler & Stice, 2001) were used to measure dysfunctional appearance beliefs. The scale measures participants’ beliefs about how important their physical appearance is in everyday domains (e.g., relationships, self-view, and emotions). For this study, and after consulting the authors of the scale, the items focusing on the perceived implications of appearance on relationships and self-views were selected and included. The reason for this approach was to minimize the number of items for an adolescent sample, but also to remove many of the age-inappropriate items (e.g., “The amount of success I have in my (future) job or career depends largely upon how I look”). The items were translated from English to Swedish by the authors of this paper. For example: “The amount of influence I have on other people depends upon how I look.” For each item, the participants indicated their level of agreement to the statements on a 5-point Likert scale, ranging from 1 = *not at all* to 5 = *extremely*. Higher scores indicate more dysfunctional appearance beliefs. Test for normality indicated that one item, “My value as a person depends upon how I look,” contributed heavily to a positive skew and kurtosis due to 71% of the sample indicating “not at all.” As another study also indicated this item as problematic (Pascoal et al., 2018), it was dropped in subsequent analyses. For the remaining eight items, internal consistency was *α* = 0.90. A one-factor CFA was then used to assess the appropriateness of using the eight items to create a latent variable (“Dysfunctional appearance beliefs”) in a measurement model. This analysis initially exposed some problems (RMSEA = 0.145; CFI = 0.899; NNFI = 0.858; SRMR = 0.050), with the most pressing being a high correlation of the residuals between some of the items. After examining the items, we determined that two pairs of items (Item 1: “The opinion others have of me is based on my appearance” vs. Item 2: “The amount of influence I have on other people depends on how I look,” and Item 7: “How I feel about myself is largely based on my appearance” vs. Item 8: “I would think more highly of myself if I looked the way I wished”) had a high degree of similarity in meaning. Consequently, we allowed the residuals from these two pairs to correlate. With these adjustments, the one-factor CFA indicated a considerably better fit (RMSEA = 0.097; CFI = 0.959; NNFI = 0.937; SRMR = 0.034).

#### Sexting with Romantic Partners and Strangers

Two questions were used to measure sexting with different sexting partners. Before answering those questions, the participants received the following definition: “Sexting is the sending and/or receiving of images or video clips that contain nudity or are sexual in nature, such as sending nude or semi-nude pictures/video clips, showing a body part, or doing a sexual act via webcam.” The first item measured sexting within a romantic relationship: “Have you sent sexting pictures/video clips of yourself to a boyfriend/girlfriend?” The second item measured sexting with a stranger: “Have you sent sexting pictures/video clips of yourself to someone you never met before?” These questions were answered with one of five response alternatives: 1 = *never*, 2 = *seldom*, 3 = *sometimes*, 4 = *often*, and 5 = *very often*. Frequency distributions for both items indicated a positive skew, with the “never” alternative the most frequent for both questions (sexting within romantic relationship = 84.9%; sexting with someone unknown = 91.9%). In line with previous studies (Rice et al., 2014; Temple et al., 2012; Van Ouytsel et al., 2014), sexting was dichotomized into 0 = “no experience of sexting” (response alternative 1), and 1 = “experience of sexting” (response alternatives 2–5).

#### Pubertal Timing

The participants’ perceived pubertal timing was measured with a global self-report question (Berg-Kelly & Erdes, 1997), translated into Swedish (Frisén & Holmqvist, 2010), asking them to evaluate their physical development relative to their age mates. The question read: “In comparison to your peers, how is your pubertal development?” and the response alternatives were: 1 = *much earlier*, 2 = *somewhat earlier*, 3 = *same*, 4 = *somewhat later*, or 5 = *much later*. Previous studies indicate that this global question is a good indicator of pubertal timing as it corresponds highly with physicians’ evaluations (Berg-Kelly & Erdes, 1997).

### Analytic Strategy

The hypothesized model proposing that pubertal timing, appearance esteem, body surveillance, and dysfunctional appearance beliefs would be linked to sexting was tested with structural equation modeling. In this model, pubertal timing was entered as a manifest predictor variable. Pubertal timing was then directly regressed on the outcome variables (i.e., sexting with a romantic partner or stranger) and also indirectly regressed on the outcome variables via the latent variables appearance esteem, body surveillance, and dysfunctional appearance beliefs. Appearance esteem, body surveillance, and dysfunctional appearance beliefs were also directly regressed on the outcome variables. For all latent variables, the corresponding item with the highest correlation to the latent variable was fixed to 1 for model identification. Appearance esteem, body surveillance, and dysfunctional appearance beliefs were allowed to correlate with each other. Age was entered as a control variable and was regressed on the two outcome variables (i.e., sexting with a romantic partner or stranger). The model was estimated using R 4.3.3 with the Lavaan package 0.6-17. Anonymized data and code are available upon request to the corresponding author.

Goodness of fit of the model was assessed using the following fit indices: RMSEA (Steiger & Lind, 1980), in which values below 0.080 were indicative of good model fit (Browne & Cudeck, 1993); CFI (Bentler, 1990), and NNFI (also known as TLI; Tucker & Lewis, 1973) with values from 0.90 to 0.95 indicating good model fit (Bentler, 1990) and values “close to” 0.95 indicating exceptional model fit (Hu & Bentler, 1999); SRMR in which values below 0.080 were indicative of good model fit (Byrne, 2006).

As the model includes two binary outcome variables, it was unsuitable to use more common estimation methods such as maximum likelihood; we opted instead for diagonally weighted least squares (DWLS; Kline, 2016). The use of DWLS requires data to be complete, and the body image variables together contained 154 (9.9% of total) missing values on at least one of the items. Listwise deletion was therefore considered unsuitable, and multiple imputation (MI) was used to replace missing values (Allison, 2001). The MI was performed using the “mice” package in R with 10 imputations and using the predictive mean matching method. Inspections also indicated that the data violated assumptions of multivariate normality, and therefore, robust standard errors were computed with mean adjusted test statistics (a.k.a. Satorra and Bentler adjustment; Satorra & Bentler, 2001). For interpretation of possible relationships in the full model with the binary outcome variables, each unstandardized regression coefficient (*b*) was exponentiated to obtain odds ratios (OR).

To determine whether the measurement model was invariant across gender, we conducted multiple group analyses in line with the steps prescribed by Byrne (2006). These steps incrementally constrained the model across the groups (equal factor loadings, equal intercepts, and equal indicator errors). If the model was invariant (ΔCFI < 0.010) across these constraints, it was assumed that the parameters behaved similarly for both genders (Putnick & Bornstein, 2016).

Finally, to formally determine any moderation effects of gender, structural invariance between girls and boys was assessed for each significant regression path. This was done by individually constraining regression paths of interest between girls and boys and determining whether this would significantly (*p* < .05) worsen the fit of the model (significant Δ *χ*^2^). The strict model (equal factor loadings, equal intercepts, and equal indicator errors) was used as reference model. If structural variance was found, it was interpreted as a significant moderation effect of gender on the specific regression path.

## Results

### Descriptive Statistics

Table 1 provides descriptive statistics and statistical tests of the group differences on the body image variables. Due to unequal variances, Welch’s *t*-tests were used, which indicated that girls reported lower appearance esteem, higher body surveillance, and higher dysfunctional appearance beliefs than boys. Effect sizes varied from medium to high. In Table 2, correlations between study variables are shown for boys and girls. In general, study variables correlated significantly with each other in the hypothesized directions. However, for boys, pubertal timing did not correlate significantly with appearance esteem nor with body surveillance. Also, very low correlations were found between appearance esteem and sexting (either recipient). Expectedly, the strongest correlations were found between the body image variables.

**Table 1:** Descriptive statistics of independent latent variables for boys and girls

Variable	Girls (*n* = 791)		Boys (*n* = 772)		Welch *t*	*df*	Mean difference (95% CI)	*d*
	*M*	SD	*M*	SD				
Body surveillance^a^	4.09	1.85	2.64	1.53	16.96*	1520.2	1.45 (1.29, 1.62)	0.85
Dysfunctional appearance beliefs^a^	2.78	1.04	2.33	0.98	8.69*	1559.5	0.44 (0.34, 0.54)	0.45
Appearance esteem^a^	2.32	0.97	2.82	0.77	− 11.32*	1496.3	− 0.50 (− 0.59, − 0.41)	0.57

^a^range: 1–5; M: mean; SD: standard deviation; df: degrees of freedom; CI: confidence interval; d: Cohens d

*p < .001

**Table 2:** Bivariate and point biserial correlations of the study variables for girls (below the diagonal) and boys (above the diagonal)

Variable	1	2	3	4	5	6	7
1. Sexting with romantic partner	–	0.56***	− 0.14***	− .05	0.23***	0.25***	0.14***
2. Sexting with stranger	0.36***	–	− 0.10**	− 0.07*	0.23***	0.23***	0.10**
3. Pubertal timing	− 0.18***	− 0.09**	–	0.03	− 0.08*	− 0.13***	− 0.08***
4. Appearance esteem	− 0.11**	− 0.08**	0.08*	–	− 0.45***	− 0.45***	− 0.07*
5. Body surveillance	0.14***	0.12**	− 0.09**	− 0.67***	–	0.68***	0.12***
6. Dysfunctional appearance beliefs	0.23***	0.19***	− 0.12***	− 0.63***	0.75***	–	0.23***
7. Age	0.27***	0.07*	− 0.11**	− 0.13***	0.13***	0.22***	–

Sexting with romantic partner and Sexting with a stranger were coded as 0: have not sent sext, 1: have sent sext

*p < .05; **p < .01; ***p < .001

### Evaluation of the Proposed Model

The full model provided good fit to the data, RMSEA = 0.053 (90% CI = 0.050 to 0.055), CFI = 0.962, NNFI = 0.962, SRMR = 0.062, *χ*^2^*(*276) = 1468.90, *p* < .001. Good model fit was also found for girls and boys in separate analyses (see Table 3). Additionally, the multiple group analysis between girls and boys showed that in comparison with the unconstrained model (i.e., free estimation of factor loadings, intercepts, and indicator errors), the imposition of equal factor loadings did not decrease fit of the CFI beyond the prescribed cutoff (ΔCFI = 0.006). Imposition of equal intercepts indicated a small CFI change (ΔCFI = 0.000), and imposition of equal indicator errors did not decrease the CFI beyond the cutoff (ΔCFI = 0.001). Thus, measurement invariance was obtained for the proposed full model, which made it suitable for between-gender comparisons (see Byrne, 2006).

**Table 3:** Test of measurement invariance of the fitted models between boys and girls

Model	χ^2^	*df*	RMSEA (CI 90%)	SRMR	NNFI	CFI	^a^Δ χ^2^	Δ*df*	ΔCFI
Gender									
Single group analysis									
Girls (*n* = 791)	753.13***	276	0.047 (0.043, 0.051)	0.062	0.971	0.971			
Boys (*n* = 772)	964.53***	276	0.057 (0.053, 0.061)	0.069	0.953	0.953			
Measurement invariance									
Equal form	1752.92***	563	0.052 (0.049, 0.055)	0.066	0.962	0.962			
Equal factor loadings	1939.31***	582	0.055 (0.052, 0.057)	0.072	0.959	0.956	42.66**	19	0.006
Equal intercepts	1953.51***	601	0.054 (0.051, 0.056)	0.072	0.960	0.956	50.53***	19	0.000
Equal indicator error	2014.25***	623	0.053 (0.051, 0.056)	0.072	0.960	0.955	97.38***	22	0.001

*df*: degrees of freedom; RMSEA: root mean square error; CI: confidence interval; NNFI: non-normed fit index; CFI: comparative fit index

^a^Satorra and Bentler (2001) scaled chi-square difference test

*p < .05; **p < .01; ***p < .001

### Paths for Girls and Boys

**Fig. 2:**
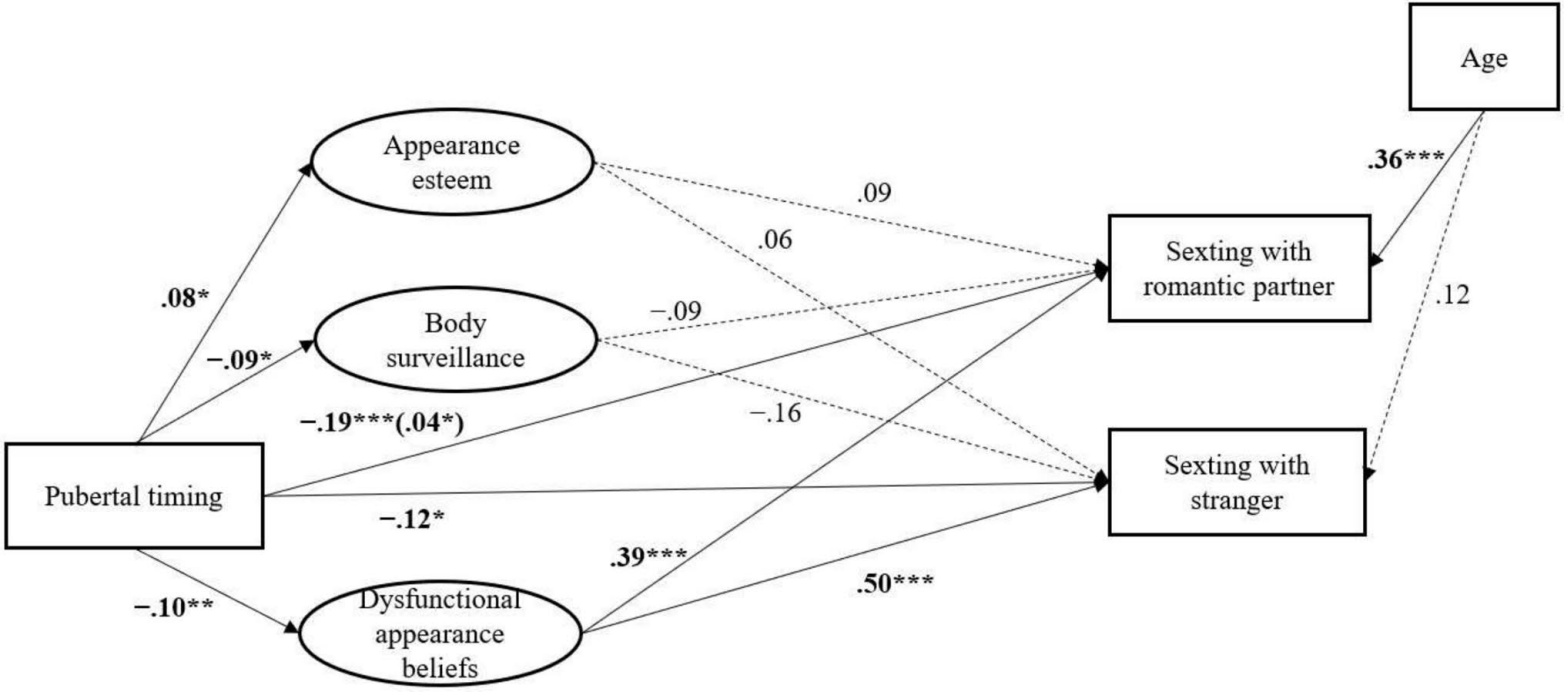
The fitted structural equation model for girls. *Note.* All regression paths in the figure are standardized. Significant paths are represented by a full line, and nonsignificant by a dashed line. The paths from the intercorrelations of appearance esteem, dysfunctional appearance beliefs, and body surveillance, as well as intercorrelations of the sexting outcomes in the model were omitted in this figure to reduce visual complexity. *p < .05; **p < .01; ***p < .001

**Fig. 3:**
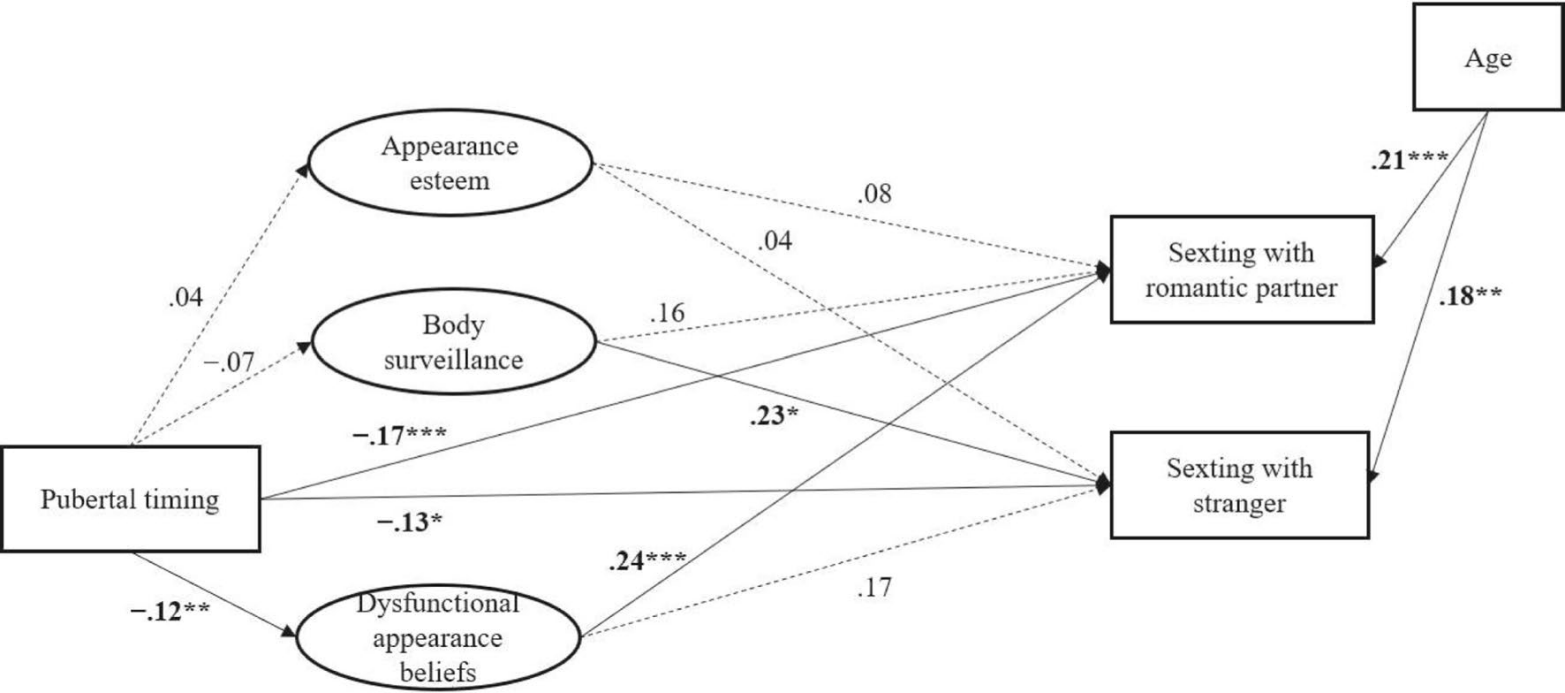
The fitted structural equation model for boys. *Note*. All regression paths in the figure are standardized. Significant paths are represented by a full line, and nonsignificant by a dashed line. Numbers within parentheses represent the indirect effect. The paths from the intercorrelations of appearance esteem, dysfunctional appearance beliefs, and body surveillance, as well as intercorrelations of the sexting outcomes in the model were omitted in this figure to reduce visual complexity. *p < .05; **p < .01; ***p < .001

Table 4 shows the direct and indirect effect of each predictor on the outcome variables. Figures 2, 3 show visual representations of the significant standardized regression paths based on the hypothetical model for girls and boys separately. Contrary to H1, we found no direct effects of appearance esteem on sexting with either recipient. Partly in line with H2, a positive direct effect of body surveillance on sexting with strangers was found, but only for boys. The strength of this effect was significantly different from the effect among girls (Δ *χ*^2^[1] = 4.69, *p* = .030). This indicates that boys who reported higher body surveillance were more likely to sext with strangers. However, the strength of this effect (OR = 1.17) was only marginally larger than the strength of the nonsignificant effect in the association between body surveillance and sexting with romantic partners (OR = 1.12). As hypothesized (H3), there was a direct positive effect of dysfunctional appearance beliefs on sexting with romantic partners for both boys and girls, indicating that participants with more dysfunctional appearance beliefs were more likely to sext with romantic partners. There was no gender difference in the strength of this effect (Δ *χ*^2^[1]) = 0.20, *p* = .650). For girls, there was also a direct effect between dysfunctional appearance beliefs and sexting with strangers, indicating that girls with more dysfunctional appearance beliefs were more likely to sext with strangers. However, there was no gender difference in the strength of this effect (Δ *χ*^2^[1] = 2.25, *p* = .130). For girls, the strength of this effect (OR = 1.43) was marginally larger than the strength of the effect in the association between dysfunctional appearance beliefs and sexting with romantic partners (OR = 1.36). For boys, the strength of the effect in the association between dysfunctional appearance beliefs and sexting with romantic partners (OR = 1.25) was marginally larger than the strength of the nonsignificant effect in the association between dysfunctional appearance beliefs and sexting with strangers (OR = 1.16). In line with H4, pubertal timing was significantly and directly associated with sexting to either recipient for both boys and girls. In other words, adolescents who reported earlier perceived pubertal timing were more likely to sext with romantic partners or strangers. The strength of these effects did not differ significantly between boys and girls (romantic partners: Δ *χ*^2^[1] = 0.15, *p* = .702; strangers: Δ *χ*^2^[1] = 0.10, *p* = .746). Partly in line with H5c, for girls pubertal timing also had a partial indirect effect on sexting with romantic partners through dysfunctional appearance beliefs. This indirect effect suggests that early-maturing girls were more likely to have dysfunctional appearance beliefs, and subsequently, were more likely to engage in sexting with romantic partners. However, when testing the strength of this effect between girls and boys, no significant difference was found (Δ *χ*^2^[2] = 0.10, *p* = .950).

**Table 4:** Effects of pubertal timing and body image factors on likelihood of sexting with romantic partners and with strangers for boys and girls

Paths	*b*	*SE*	*β*	OR (95% CI)
*Sent sext to romantic partner*				
Girls				
Pubertal timing (DE)	− 0.23***	0.06	− 0.20	0.79 [0.71, 0.88]
Pubertal timing → Appearance esteem (IE)	0.01	0.01	0.01	1.01 [0.99, 1.03]
Pubertal timing → Body surveillance (IE)	0.01	0.02	0.01	1.01 [0.98, 1.04]
Pubertal timing → Dysfunctional appearance beliefs (IE)	− 0.05*	0.02	− 0.04	0.95 [0.91, 0.99]
Pubertal timing (TE)	− 0.26***	0.06	− 0.22	0.77 [0.69, 0.86]
Appearance esteem (DE)	0.08	0.09	0.09	1.08 [0.91, 1.29]
Body surveillance (DE)	− 0.05	0.09	− 0.09	0.95 [0.80, 1.12]
Dysfunctional appearance beliefs (DE)	0.31**	0.10	0.39	1.36 [1.11, 1.68]
*R*^2^ = 0.27				
Boys				
Pubertal timing (DE)	− 0.18**	0.06	− 0.17	0.83 [0.74, 0.93]
Pubertal timing → Appearance esteem (IE)	0.00	0.01	0.00	1.00 [0.99, 1.01]
Pubertal timing → Body surveillance (IE)	− 0.01	0.01	− 0.01	0.99 [0.97, 1.01]
Pubertal timing → Dysfunctional appearance beliefs (IE)	− 0.03	0.02	− 0.03	0.97 [0.94, 1.00]
Pubertal timing (TE)	− 0.23***	0.07	− 0.20	0.79 [0.71, 0.90]
Appearance esteem (DE)	0.11	0.10	0.08	1.12 [0.92, 1.35]
Body surveillance (DE)	0.11	0.07	0.16	1.12 [0.97, 1.27]
Dysfunctional appearance beliefs (DE)	0.22*	0.09	0.24	1.25 [1.04, 1.48]
*R*^2^ = 0.20				
*Sent sext to stranger*				
Girls				
Pubertal timing (DE)	− 0.13*	0.06	− 0.12	0.88 [0.76, 0.98]
Pubertal timing → Appearance esteem (IE)	0.01	0.01	0.01	1.01 [0.99, 1.03]
Pubertal timing → Body surveillance (IE)	0.02	0.02	0.01	1.02 [0.93, 1.02]
Pubertal timing → Dysfunctional appearance beliefs (IE)	− 0.05	0.03	− 0.05	0.95 [0.91, 1.00]
Pubertal timing (TE)	− 0.17**	0.07	− 0.15	0.84 [0.74, 0.96]
Appearance esteem (DE)	0.06	0.09	0.06	1.06 [0.88, 1.26]
Body surveillance (DE)	− 0.09	0.10	− 0.16	0.91 [0.76, 1.12]
Dysfunctional appearance beliefs (DE)	0.36**	0.14	0.50	1.43 [1.10, 1.88]
*R*^2^ = 0.16				
Boys				
Pubertal timing (DE)	− 0.15**	0.06	− 0.13	0.86 [0.77, 0.97]
Pubertal timing → Appearance esteem (IE)	0.00	0.00	0.00	1.00 [0.99, 1.01]
Pubertal timing → Body surveillance (IE)	− 0.02	0.01	− 0.02	0.98 [0.96, 1.01]
Pubertal timing → Dysfunctional appearance beliefs (IE)	− 0.02	0.01	− 0.02	0.98 [0.95, 1.01]
Pubertal timing (TE)	− 0.19**	0.07	− 0.17	0.83 [0.75, 0.94]
Appearance esteem (DE)	0.06	0.11	0.04	1.06 [0.86, 1.31]
Body surveillance (DE)	0.16*	0.07	0.23	1.17 [1.02, 1.33]
Dysfunctional appearance beliefs (DE)	0.15	0.09	0.17	1.16 [0.97, 1.39]
*R*^2^ = 0.19				

*b*: unstandardized regression path; *SE*: standard error; *β*: standardized regression path; OR: odds ratio; CI: confidence interval; DE: direct effect; IE: indirect effect; TE: total effect

*p < .05; **p < .01; ***p < .001

For age (control variable), we found a significant positive association with sexting with romantic partners among girls (*b* = 0.45, *SE* = 0.07, *β* = 0.36, OR (95% CI) = 1.57 [1.38, 1.79]), but not with sexting with strangers (*b* = 0.14, *SE* = 0.08, *β* = 0.12, OR (95% CI) = 1.15 [0.98, 1.35]). This indicates that older girls were more likely to sext with romantic partners. Among boys we found significant positive associations between age and sexting with romantic partners (*b* = 0.24, *SE* = 0.07, *β* = 0.21, OR (95% CI) = 1.27 [1.11, 1.45]), and with strangers (*b* = 0.21, *SE* = 0.08, *β* = 0.18, OR (95% CI) = 1.23 [1.06, 1.44]), indicating that older boys were more likely to sext with both romantic partners and strangers. When testing gender differences in the strength of the effect of age, we found no significant differences for sexting with romantic partners (Δ *χ*^2^[1] = 2.08, *p* = .150), or sexting with strangers (Δ *χ*^2^[1] = 0.94, *p* = .333).

## Discussion

The overarching aim of the present study was to examine the links between pubertal timing and different body image factors with adolescents’ sexting behaviors. We also investigated whether relations between body image factors and sexting differed depending on the recipient of the sext (romantic partner or stranger), and whether patterns in the results differed between boys and girls. Contrary to our hypothesis (H1), and contrary to other studies (Bianchi et al., 2017; Howard et al., 2021), the link between appearance esteem and sexting to either partner was not significant for girls or for boys. For body surveillance, we found a link to sexting with strangers—but only for boys which only partly supports our second hypothesis (H2). For dysfunctional appearance beliefs, the proposed links were fully supported regarding sexting with both romantic partners and strangers among girls (H3). For boys, only partial support was found as dysfunctional appearance beliefs was only linked with sexting with romantic partners, but not with strangers. As predicted (H4), both early-maturing girls and boys were more likely to engage in sexting with any partner, which is consistent with previous studies (e.g., Burén & Lunde, 2018). Interestingly, and only for girls, there was an indirect link between early pubertal timing and sexting with romantic partners via dysfunctional appearance beliefs. This link only partly supported our fifth hypothesis (H5c) and was the only significant indirect effect that was found. Furthermore, we found only marginal differences in the strength of the effects in the associations between the body image factors and the two sexting outcomes—sexting with a romantic partner or a stranger—for both boys and girls.

### Appearance Esteem and Sexting

Although appearance esteem correlated with sexting with both recipients in the hypothesized directions, appearance esteem was not significantly associated with boys’ or girls’ sexting in the proposed model. The age of our participants, who are younger than those in previous studies, might have contributed to this result. During early adolescence, appearance esteem undergoes significant changes, with both girls and boys becoming increasingly dissatisfied with their physical appearance (Frisén et al., 2015). Indeed, similar research has shown lower levels of appearance esteem being linked with more sexting in slightly older samples (Bianchi et al., 2017; Howard et al., 2021). It is also possible that the link between appearance esteem and sexting behaviors is more complex than was accounted for in our proposed model. For instance, although we hypothesized that less appearance esteem would be associated with more sexting with either recipient, the effect could also be positive, at least for some adolescents. Some adolescents with higher appearance esteem might be more comfortable with their physical appearance, making them more inclined to sext. Although the small but significant correlations between appearance esteem and sexting indicated a negative relationship (lower appearance esteem was associated with sexting), analyses that are designed to shed light on within-group differences might yield more nuance. Furthermore, distinguishing between state and trait appearance esteem could help clarify these relationships. We believe the notion proposed by Howard et al. (2019) that state body satisfaction may be involved in decisions about sexting, is an interesting avenue for future research. Investigating how adolescents who engage in sexting perceive their body and appearance just before and after sexting situations could provide valuable insights.

### Body Surveillance and Sexting

We found that boys who self-monitor their appearance (body surveillance) were more likely to engage in sexting with strangers. These findings align with Liong and Cheng’s (2019) findings, demonstrating associations between body surveillance and sexting motives, as well as sexting behaviors among young people. However, unlike this previous study where this associations were observed for both genders, our study only found this link among boys. One possible explanation for this discrepancy is that by including dysfunctional appearance beliefs in the model and differentiating the recipient of the sexts, we were able to uncover more nuanced results that highlight gender differences. This is a novel finding that enhances our limited understanding of boys' sexting, and one explanation could be that boys who engage in body surveillance, sexting with strangers may serve as an outlet for constant self-monitoring, allowing for comparisons and seeking external appearance validation. The reason why this does not seem to apply when sexting with romantic partners is possibly due to the increased stakes associated with sending explicit images to a romantic partner, such as the risk of a negative reputation in school or repercussions from adults (Doyle et al., 2021; Ringrose et al., 2012). However, it's important to acknowledge that these arguments are speculative, and further studies investigating the role of body surveillance in boys' sexting are needed. For instance, qualitative in-depth interviews could provide insights into how body surveillance may drive boys’ motivations, experiences, and perceptions of sexting.

### Dysfunctional Appearance Beliefs and Sexting

A main and novel finding of this study was that girls and boys who reported more dysfunctional appearance beliefs were more likely to engage in sexting with romantic partners. This finding aligns with the cognitive-behavioral framework and the theoretical notion of behaviors resulting from being appearance schematic, that is, highly invested in physical appearance (Cash, 2012). As emphasized by Spangler and Stice (2001), having dysfunctional appearance beliefs means the adolescent believe that physical appearance has important implications in everyday domains which makes the individual much more attentive to appearance-related stimuli and creates an exaggerated focus on one’s body and physical appearance for self-perception and self-evaluation. Thus, our study may show that the threshold for engaging in sexting with romantic partners may be lower for adolescents who score high on dysfunctional appearance beliefs, as they may hold a strong belief that displaying their body to others comes more natural in sexual interactions. Indeed, previous studies indicate that one motive for sexting is to maintain romantic relationships (Lenhart, 2009; Lippman & Campbell, 2014), and to gain popularity (Del Rey et al., 2019), which may be especially important for young people. The fact that dysfunctional appearance beliefs were associated with girls’ sexting not only with romantic partners but also with strangers, may indicate that these beliefs may be an important factor for girls in sexting situations regardless of the relationship status with the other person. Although the quality or specific circumstances of the sexting situations were not examined in this study, we know from previous studies that many adolescents, especially girls, can feel pressure to sext (Burén & Lunde, 2018; Reed et al., 2020). It may well be the case that girls who hold strong beliefs that appearance is important in social relationships are among those who are more likely to give in to this pressure, regardless of whom they are sexting with. However, these suggestions remain speculative, and it should be stressed that we are unaware of whether the adolescents in this study experienced their reported sexting experiences as positive or negative. Furthermore, it is important to note that despite its name, dysfunctional appearance beliefs do not necessarily imply that sexting for this reason is solely for negative purposes. This construct reflects an exaggerated perception that physical appearance holds significant implications in everyday domains and interpersonal relationships. Many adolescents undoubtedly share this perception, and their reasons for sexting could instead be to deepen romantic relationships or flirt with others.

This study revealed a mediation effect involving dysfunctional appearance beliefs in the relationship between pubertal timing and sexting behavior with romantic partners. Specifically, the findings suggest that, among girls, early pubertal timing was associated with higher levels of dysfunctional appearance beliefs, and these beliefs were in turn associated with a greater likelihood of engaging in sexting behavior with romantic partners. This finding may mirror the greater sex-appeal self-worth previously reported among early-maturing girls (Grower et al., 2019). Early-maturing girls may have a greater interest for sexual activities compared to their female peers, but it may also be that early-maturing girls are socialized into the belief that their body is an important sexual asset (see self-objectification theory, Fredrickson & Roberts, 1997). One can only speculate as to why this only applies when they are sexting with a romantic partner and not a stranger, but one possible reason is that when engaging with familiar individuals, it may be perceived as more important to present oneself in accordance with sexualized ideals of women. Another plausible explanation could be that some adolescents feel more comfortable presenting themselves in a sexualized manner to a romantic partner, thus increasing the likelihood of sexting. However, since we found no significant differences between girls and boys in the indirect effect of dysfunctional beliefs in the association between pubertal timing and sexting with romantic partners, and observed only a small effect among girls, this result should be interpreted with caution.

### Strengths and Limitations

Although the present study provides a novel contribution to the literature by focusing on the role of different dimensions of body image for adolescents sexting behaviors, some limitations merit extra attention. First, the present study used a cross-sectional approach, which precludes inferences about causality. Here it could be noted that it is not ideal to test mediation with cross-sectional data as it by nature captures information at a single point in time. However, given the empirical evidence underlying the assumption that pubertal timing precedes both body image issues (e.g., Hamlat et al., 2015) and sexual behaviors (Skoog et al., 2009), it was deemed a suitable approach. Although the hypothesized model was theoretically and empirically driven, the direction of effects may be different from what we assumed. In fact, one may expect that the relations between body image factors and sexting are bidirectional, meaning that body image and sexting may affect each other. For instance, adolescents who think physical appearance is important for relationships and engage in sexting may have this belief reinforced by feedback from the one receiving the sext.

Second, although the aim of this study was to examine adolescents’ actual sexting behavior, and not their motivations or attitudes to sexting as has been done in previous research (e.g., Le et al., 2023), these aspects (motivations and attitudes to sexting) could have been explored together with sexting behavior in a more comprehensive model. By also including motivational aspects of sexting, we perhaps would have been able to clarify the relationships between appearance esteem with sexting. This would have helped to better understand the association between body image and motivational processes underlying adolescent sexting.

Third, the sexting variables were somewhat limited as they lacked information on whether the participant actually perceived opportunities or risks when sexting with a romantic partner or stranger. Although the outcome variables were chosen based on the argument, derived from previous research (e.g., Dowdell, 2011; Fleming & Rickwood, 2004; Livingstone et al., 2011; Rice et al., 2014; Williams et al., 2013), that sexting with known versus unknown others may differ in terms of determining factors and risks, we cannot tell from the present study if sexting with romantic partners or strangers represents different levels of risk for adolescents. It should also be noted that there was an overlap between the outcome variables, showing that adolescents (about 1/3 girls and 1/10 boys) who sexted with a romantic partner also sexted with strangers. Future studies would benefit from using more refined measures of sexting, considering both the quality of the sexting experiences and the potential accumulated risks of sexting with many different recipients.

Fourth, the present study could also have included other body image measures, such as measures of positive body image. Although higher levels of appearance esteem is an indicator of body satisfaction, including a body image measure conceptually measuring positive body image would have shed light on whether and how more comprehensive features of positive body image (e.g., body acceptance, body appreciation, functional appreciation, and broad conceptualization of beauty; Alleva et al., 2017; Tylka & Wood-Barcalow, 2015) are associated with sexting in our study. Indeed, a recent study found that low levels of body appreciation was associated with more sexting among adolescents (Paquette et al., 2022), which shows the importance of including positive aspects of body image. Also, examining adolescents’ experiences of embodiment (Piran et al., 2020), including their experiences of connection with their body and attunement to sexual desires, could help to further disentangle associations between adolescents’ relation with their bodies and their propensity to sext. It is also possible that both negative and positive body image and experience of embodiment can co-occur, making the associations with sexting even more complex.

Fifth, the measurement models of the body image scales indicated some issues. Specifically, although the CFI, NNFI, and SRMR indicated good fit, the RMSEA for the scales was above 0.080. While it could be argued that the values fell within the range often deemed acceptable (< 0.100) in SEM-analyses (MacCallum et al., 1996), there is a possibility that the scales may not fully have captured the underlying constructs of interest with optimal precision. This issue could stem from various sources, such as large un-accounted measurement errors or model misspecification. Nevertheless, caution is warranted when interpreting the results derived from these scales, as the relatively high RMSEA value may introduce some degree of uncertainty into the findings.

Finally, in this study, we solely focused on visually based sexting. However, one could argue that text-based sexting also has the potential to raise body awareness. For example, comments describing specific body parts may draw undue attention to those parts for the recipient. Studies indicate that appearance-based comments, even if positive, on social media are linked to heightened body awareness and the risk of body dissatisfaction (e.g., Fatt & Fardouly, 2023; Tiggemann & Barbato, 2018). Similar experiences might hold true for text-based sexting, suggesting an interesting area for future research concerning the relationship between sexting (both visually and text-based) and body image.

### Conclusions

The present study supports that body image factors are associated with adolescents’ sexting experiences. In particular, dysfunctional beliefs about appearance were linked with adolescents’ sexting with both romantic partners and strangers. Body surveillance was also associated with sexting with strangers, but only for boys. Early pubertal timing was associated with an increased likelihood for sexting with either sexting partner among both girls and boys, and for girls, dysfunctional appearance beliefs mediated this link. Unexpectedly, for both girls and boys, no significant association between appearance esteem and sexting was found. The findings of this study could inform future studies into the complexities of how different aspects of body image are associated with sexting. Aspects such as the quality of sexting experiences, the role of positive body image, and the causal pathways between body image and sexting behaviors are all interesting areas for future research.

More specifically, our results point to the importance of integrating discussions on body image and sexting into comprehensive sexuality education curricula tailored to girls and boys respectively (see Ojeda & Del Rey, 2022). Healthcare providers and policymakers can also use these findings to advocate for comprehensive sexual health policies that prioritize informed consent, communication skills, and respect in sexting interactions. By emphasizing the importance of autonomy in sexual decision-making, stakeholders can promote positive and safe sexting while also mitigating potential risks associated with sexting. Ultimately, adolescents should be empowered to engage in sexting only when it is a matter of volition and choice, and they feel genuinely comfortable doing so.

## Data Availability

The data that support the findings of this study are available from the corresponding author upon reasonable request. The data that support the findings of this study are available on request from the corresponding author after standard confidentiality assessment required by Swedish law ("Public Access to Information and Secrecy Act" 2009:400). The data are not publicly available due to that ethics approval does not include open sharing of raw data as it contains personal information that may lead to participant identification.
